# Therapeutic Drug Monitoring Is a Feasible Tool to Personalize Drug Administration in Neonates Using New Techniques: An Overview on the Pharmacokinetics and Pharmacodynamics in Neonatal Age

**DOI:** 10.3390/ijms21165898

**Published:** 2020-08-17

**Authors:** Domenico Umberto De Rose, Sara Cairoli, Marco Dionisi, Alessandra Santisi, Luca Massenzi, Bianca Maria Goffredo, Carlo Dionisi-Vici, Andrea Dotta, Cinzia Auriti

**Affiliations:** 1 Neonatal Intensive Care Unit, Department of Medical and Surgical Neonatology, “Bambino Gesù” Children’s Hospital IRCCS, 00165 Rome, Italy; derosedomenicoumberto@gmail.com (D.U.D.R.); alessandra.santisi@opbg.net (A.S.); andrea.dotta@opbg.net (A.D.); 2 Laboratory of Metabolic Biochemistry Unit, Department of Specialist Pediatrics, “Bambino Gesù” Children’s Hospital IRCCS, 00165 Rome, Italy; sara.cairoli@opbg.net (S.C.); marco.dionisi@uniroma1.it (M.D.); biancamaria.goffredo@opbg.net (B.M.G.); carlo.dionisivici@opbg.net (C.D.-V.); 3 Neonatal Intensive Care Unit and Neonatal Pathology, Fatebenefratelli Hospital, 00186 Rome, Italy; luca.massenzi@fbf-isola.it

**Keywords:** therapeutic drug monitoring, personalized medicine, neonates, newborns, infants, pharmacokinetics, pharmacodynamics, drugs

## Abstract

Therapeutic drug monitoring (TDM) should be adopted in all neonatal intensive care units (NICUs), where the most preterm and fragile babies are hospitalized and treated with many drugs, considering that organs and metabolic pathways undergo deep and progressive maturation processes after birth. Different developmental changes are involved in interindividual variability in response to drugs. A crucial point of TDM is the choice of the bioanalytical method and of the sample to use. TDM in neonates is primarily used for antibiotics, antifungals, and antiepileptic drugs in clinical practice. TDM appears to be particularly promising in specific populations: neonates who undergo therapeutic hypothermia or extracorporeal life support, preterm infants, infants who need a tailored dose of anticancer drugs. This review provides an overview of the latest advances in this field, showing options for a personalized therapy in newborns and infants.

## 1. Introduction

“Children are not small adults”: if this statement is true for pediatric medicine, it is certainty true in the fields of pharmacokinetics and pharmacodynamics in children. More recent studies on these topics force us to complete this sentence, stating that “infants are not just small children”. In humans, organs and metabolic pathways undergo deep and progressive maturation processes after birth. When studying neonatal pharmacology, we must take this into account [1]. Almost 65% of the drugs used to treat newborns in the Neonatal Intensive Care Units (NICUs) are off-label and need optimization in dosage administration still now. TDM could help to optimize drug exposure for clinical efficacy and to prevent toxicity [2]. Therefore, therapeutic drug monitoring (TDM) should be adopted in NICUs, where the most preterm and fragile babies are hospitalized and treated with many drugs [3]. This review provides an overview of the latest advances in this field, showing options for a personalized therapy in this delicate patient population.

## 2. Developmental Changes in Newborns

Interindividual variability in response to drugs is well known in both adults and children. Different factors are involved in this variability. While in adults the development of cells, tissues and organs involved in drug metabolism is complete, in neonates their functional development still continues [4]. Two key parameters, the volume of distribution (Vd: the theoretical volume of liquid in which the totality of the administered drug should be diluted to produce the plasma concentration) and the clearance (Cl: the volume of plasma from which a substance is completely removed per unit time), well describe the pharmacokinetics (PK) of a given compound. We should also consider the bioavailability (F: percent area under the curve compared to the intravenous route where F is 1, by definition) and the absorption rate [5], when the route of administration is not intravenous. These three parameters depend on absorption, distribution, metabolism, and excretion (ADME).

**Figure 1:**
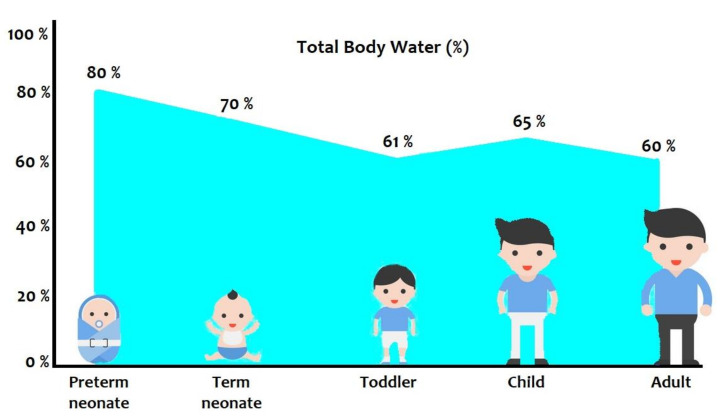
Body composition (total body water) and growth. Modified from Rodieux et al. [11].

1. **Absorption**. When the drug administration is by non-intravenous route, the absorption depends on various patient-related factors, due to functional maturation processes of organs and systems. If the drug is administered orally, its absorption depends on gastric emptying, gastric pH, intestinal motility, intestinal first pass metabolism and permeability [5]. Moreover, the composition of milk (formula, hydrolysate, or human milk) could influence gastric emptying, which is usually slower in neonates than in children [6]. Concomitant medications, such as proton pump inhibitors, may reduce or alter gastric pH [7]. Congenital defects, such as duodenal/ileocolic atresia or stenosis, and bowel diseases, such as necrotizing enterocolitis [8] associated with post-surgery short bowel syndrome, may alter the development of intestinal transporters (i.e., PEPT1) [9], further influencing the variability of drugs absorption.
2. **Distribution**. The body composition is a key factor in drug distribution in individuals. Neonates, in particular preterm neonates, have body fat stores lower and total body water (TBW) higher than children and adults (Figure 1) [10,11]. These characteristics have a deep impact on the distribution volume of both lipophilic (lower distribution volume: e.g., diazepam and propofol) and hydrophilic (higher distribution volume: e.g., aminoglycosides and paracetamol) compounds in neonates. The protein binding of drugs also influences their distribution in the body. Compared to adults, neonates have lower concentrations of most plasma binding proteins (e.g., albumin, α1-acid glycoprotein, or plasma globulins). Furthermore, in newborns, an increase of concentrations of bilirubin and free fatty acids could be observed, and this can result in a competitive binding of the drugs to albumin [12,13].
  At birth, human serum albumin (HSA) concentrations are close to adult levels (75–80%), while α1-acid glycoprotein (AAG) is initially half the adult concentration: therefore, drug clearance in neonates seems to be higher for AAG-bound drugs (e.g., fentanyl and propranolol) than for HSA-bound drugs (e.g., beta-lactams and morphine) [14]. In particular, the low levels of albumin reduce the fraction of drugs strongly linked to albumin, causing the increase of the free fraction and therefore of the elimination of the drug. The low levels of α-1 acid glycoprotein cause an increase in apparent Vd and/or an increase in toxicity.
  Therefore, changes in body composition and in the ability to bind drugs with proteins, typical of the individual’s growth and maturation process, modify deeply the distribution of drugs in the body. Although the percentage of TBW does not change significantly after one year of age, a progressive decrease in extracellular water occurs from childhood to young adulthood, having a significant effect on the pharmacokinetics of drugs.
3. **Metabolism**. The rapid maturation process of the neonatal liver affects many biotransformation functions. The enzymes expression varies, from the fetal and postnatal period to one year of age, when it reaches almost complete functional activity [15]. Enzymes responsible for drugs metabolism are divided into Phase I enzymes, which are involved with primary oxidation, reduction and hydrolysis processes, and Phase II enzymes, which are responsible for conjugation of drug compounds to allow excretion [16]. Most important Phase I enzymes belong to the cytochrome (CYP) 450 family (CYP1A2, CYP2D6, CYP2C9, CYP2C19, CYP3A4 and CYP2E1) [10,17]. Phase II enzymes include glucuronidation, sulfation, methylation and acetylation [10]. The hepatic expression of cytochrome enzymes is characterized by dynamic modifications and a marked interindividual difference, affecting the metabolism of many drugs and their efficacy [18].
  Although the liver is the major site of drug metabolism, the kidneys can also be involved, but data on developmental processes of renal microsomal systems are still lacking [19].
4. **Excretion**. Drug excretion is usually via renal or hepatic routes. Preterm neonates prior to 36 weeks of gestation reveal a still ongoing nephrogenesis, which continues after birth [20]. Neonatal glomerular filtration rate (GFR) is low at birth and increases from 10–20 to 30 mL/min/1.73 m^2^ after two weeks. Levels of GFR (120 mL/min/1.73 m^2^) normally observed in adults are reached around two years of age [21]. Serum creatinine levels are elevated at birth (60–70 μmol/L), reflecting maternal values; GFR and the tubular reabsorption of creatinine are still low. Moreover, serum creatinine clearance (CrCl) increases with gestational age, especially in extremely low birth weight (ELBW) infants [22]. The age at which preterm infants reach renal function similar to that of term infant is poorly known. Low GFR delays drugs clearance, prolongs their half-lives and these effects are more pronounced in preterm infants [21]. Similar to renal excretion, the phenotypic variation in drugs hepatic metabolism and excretion also depends on constitutional, disease-related and genetic factors [23].

Dosing regimens for neonates are usually empirically derived from adult experiences, using linear extrapolation based on body weight. It is therefore very difficult to get the large number of blood samples required to produce individual PK models from so fragile babies [9]. However, developmental changes are dynamic processes: this physiological characteristic of neonates can lead to therapeutic failures and unsafe side effects during the therapy [24]. Moreover, there are many interactions between genetic factors, which influence pharmacokinetics and pharmacodynamics (e.g., genetic polymorphisms of cytochrome CYP2C19 that influence metabolism of benzodiazepines and proton pump inhibitors), as well as environmental factors (e.g., a still ongoing nephrogenesis, eventual malformations or injuries and the administration of concomitant drugs, especially in preterm neonates) [13].

All these factors play a role in the exposure and response to drugs. There is still now a significant gap between knowledge in genetics and practical application for modeling of drug profiles on the genotype of the subject [4].

Even if patients receive the same drug doses, a different response could be obtained: the first objective of the Therapeutic Drug Monitoring (TDM) is to measure plasma drug concentrations to adjust and personalize the drug dosage and administration [25]. Not all drugs require TDM. It is conversely crucial in specific circumstances: the narrow range between lethal dose and therapeutic dose, to verify the therapy compliance of the patient, when there is a high variability inter or intra patient, or to prevent too high serum levels and unsafe effects. In neonates, every single week of life has its physiological characteristics and should be considered as a world in itself. Neonates exhibit a higher variability in drug exposure, drug efficacy and toxicity than adults. For all these reasons, neonates frequently need TDM [26]: it still appears as a feasible and cost-effective instrument in this special population [27], and the number of drugs requiring TDM is increasing.

## 3. Sampling and Alternative Specimens

Most TDM assays measure blood or plasma drug concentration on blood samples, picked up by venipuncture. Depending on the instrument characteristics, a satisfying amount of blood is necessary for measurements. Doing TDM in neonates implies ethical and clinical considerations, as venipuncture is distressing and painful in this fragile population. Moreover, frequent, and/or extensive sampling should be avoided, because of the limited total blood volume of neonates, the risk of iatrogenic anemia and the risk of blood transfusions. In the last decades, different sampling methods have been proposed to limit blood loss and currently non-invasive methods and more reliable matrices are recommended in PK study and TDM practice [28]. To overcome clinical and ethical issues of blood drawing, several microsampling approaches have recently been developed.

Since the 1960s, dried blood spot (DBS) has been used for metabolic screening test in neonates, recovering sample through heel stick. DBS cards consist of filter paper, in which whole blood is blotted in a premarked spot. Then, after an extraction phase, DBSs are dried at room temperature and sent to laboratory for analysis. The user-friendliness and minimal invasiveness make DBS an attractive sampling method for TDM purpose in neonates [29]. DBS sampling has been validated and applied in newborns to assess concentration of several drugs, such as antibiotics, corticosteroids, and caffeine, for both therapeutic drug monitoring and PK studies application [30,31,32,33]. However, this method presents some limitations. The most critical limit is the variability related to the hematocrit value. Differences in viscosity may also affect the blood volume on the spot, increasing the probability of assay inaccuracies. Moreover, the punch location to obtain the blood sample is affected by the inhomogeneity of drug on the filter paper. Given those limitations, TDM on DBS samples requires further validation, especially when applied to quantitative bioanalysis [34,35].

Capillary microsampling (CMS) allows collecting minimal blood volume by microtubes devices. Currently, this method is spreading in preclinical study in accordance with 3R principles [36]. In addition, CMS has been coupled with DBS to overcome the volume variability in the collection of blood samples. However, capillary devices also represent a promising approach for TDM in neonates [37]. As an example, in a comparative PK study on neonates, our group has successfully used microtubes to collect capillary blood samples using a heel stick, overcoming phlebotomy [38]. Furthermore, CMS is currently used in our institution for routine monitoring of antibiotics in critically ill neonates [39].

Volumetric Absorptive Microsampling (VAMS) device consists in a plastic handler with a hydrophilic tip, which is used to sweep up liquid matrices such as blood, saliva, urine, sweat and tears. After a drying step, the sample is stored in a clamshell, sent to laboratory and recovered with an extraction solvent. To date, VAMS is still a novel microsampling method and is not yet routinely used in neonates. Since first data published in 2014 [40], several drugs have been monitored through VAMS such as antiepileptics, anthelmintic and immunosuppressants [41,42,43,44]. VAMS can be utilized to collect blood samples from a single finger or heel prick, allowing to obtain accurate volumes, without hematocrit’s influence. Nevertheless, caution should be taken to avoid the excess of blood absorbed on the tip, which could lead to specimen volume variability. In our opinion, VAMS represents an easy-to-use alternative to DBS and capillary heel stick. Currently, our group is investigating the application of VAMS for TDM purpose in pediatric patients, including neonates. Preliminary data on antiepileptic drugs have shown an acceptable plasma/VAMS correlation of drug concentration (work of our group still in progress).

Even though reference ranges have been established in plasma or blood, the use of alternative matrices represents another option to perform TDM in neonates, make the sampling process easy and avoid pre-analytical bias.

Recently, saliva testing is becoming a promising approach in diagnosis and progression of some diseases [45]. Moreover, if saliva/plasma ratio is close to 1, salivary concentration could provide reliable results for TDM. Many physiological and pathological factors affect drug exchange between oral fluids and plasma. However, dissociation constants of drug and salivary pH are primarily involved in this process. Indeed, neutral, and very weakly acidic drugs showed an excellent correlation between saliva and plasma [46]. Although collecting samples through spitting into tubes is the simpler method in adults and children, this is challenging in neonates. Thus, various techniques have been described, such as the use of pipettes, adapted pacifiers and other specifically designed devices [47,48].

Typically, urine analysis has been considered the primary choice for qualitative analysis, especially for toxicological screening. The adequate specimen volume and the accumulation of many drugs and metabolites make this matrix a good option for TDM. On the other hand, patient hydration, urinary pH, renal function and diuretic comedication lead to analytical issues [49]. Care must be taken in analyzing light sensitive compounds, due to the longer exposure to daylight and the reduced turbidity, compared to blood. To date, quantitative analysis in urine matrix should be more thoroughly investigated and can be considered only for the assessment of therapy adherence [50].

A sampling technique that integrates the use of microsampling with alternative matrices analysis is microdialysis. It consists in a semipermeable catheter, able to separate permeable molecules within the matrix. Microdialysis has been used in neonates for continuous monitoring of endogenous analytes such as glucose or lactate, especially in ICU setting [51,52]. However, it could be applied only for research purposes until now [53].

To date, the blood/plasma dogma is beginning to lose its grip in TDM for neonates, mainly because of clinical, ethical, and analytical drawbacks related to this vulnerable population. Nevertheless, further investigation on simpler and more reliable sampling strategies are needed, despite hopeful approaches have been developed.

## 4. Drug Monitoring Methods

A crucial point of TDM is the choice of the bioanalytical method to measure drug concentration in plasma or in other biologic fluids. Two of the most used techniques are: immunoassays and mass spectrometry (MS) coupled by high performance liquid chromatography (LC) or gas chromatography (GC). The question all experts try to answer is: “Immunoassay or mass spectrometry, which is the best?” [54].

Immunoassays are based on the binding of an antigen to an antibody. This reaction is reversible and depends on the antigen and antibody concentrations, antibody’s affinity for the antigen, temperature, pH and other parameters. Through this link immunoassays methods can identify and quantify analytes in biologic matrices. Immunoassays are captivating for clinical applications in routine analysis, especially for TDM. Advantages of this technique are many: it is easy to work, allows an automation system, is reproducible between different laboratories and works by validated kits [54,55]. On the other hand, immunoassay shows some limitations that could hamper its TDM application: low specificity, due to reaction of cross-reactivity among parental and drug metabolites; significant between-assays imprecision that could lead to an erroneous interpretation of drug concentration; and huge variability of lower limit of detection between different immunoassay analytical methods [55,56,57,58,59,60,61].

MS coupled with high-performance LC (LC-MS/MS) is an analytical technique to quantify substances and identify unknown compounds. LC systems through chromatographic column allow separating compound in relation to polarity and affinity to the column. Mass spectrometer generates multiple ions from the sample and separates these ions according to their specific mass-to-charge ratio, recording relative abundance of each ion type [56]. LC-MS/MS represents now the gold standard for TDM [25] and offers many advantages in its application in neonatal TDM: sensitivity, precision and accuracy, even with small amounts of samples, as the technique is very sensitive with small concentrations of analytes [2,25]. The benefits of MS are speed of analysis, simple preparation of samples and multiple analytes’ determination in one short run [54].

An important aspect for the application of LC-MS/MS method is validation by guidelines of the European Medicines Agency (EMA) [62] and the Food and Drug Administration [63]. Different points are being investigated for validation method: precision, accuracy, selectivity for endogenous and exogenous substances, carryover, matrix effect, recovery, stability, dilution integrity and calibration standard range on the basis of concentration expected in clinical samples [57].

Last year, Shipkova et al. described a new approach of TDM: pharmacodynamics monitoring as an integral part of TDM. Pharmacodynamic (PD) TDM based on biomarkers is possible by new instrumentation: mass spectrometer, computational biology and pharmacology and bioinformatics database. Use of these new technologies to quantitative measurements of biochemical process can give information of interaction between drug and target. These integrated approaches to PK-PD monitoring by TDM allow having a complete clinical view [59].

## 5. Excipients

Excipients are essential components of drug formulations to overcome challenges such as solubility and stability. Excipients may also bear potential toxicity in pediatric population and safety risk should be considered when they are administered in neonates.

Excipient exposure is related also to route of administration and gestational age. Parenteral drugs, often produced as single dose vials where preservatives can be avoided, contain fewer excipients than topical and enteral formulations [64]. However, accepted daily intakes (ADIs) are often exceeded also with intravenous formulations [65]. The knowledge on the safety or toxicity of excipients in neonates is limited still now and European Study of Neonatal Exposure to Excipients (ESNEE) [64] and Safety and Toxicity of Excipients for Paediatrics (STEP) database initiatives [66] are carrying on, focused on clinical pharmacology of excipients in neonates. A first policy could consist in replacement of the most commonly used drugs with similar excipients-free active pharmaceutical ingredients that may spare up to 44% of neonates from exposure to potentially harmful excipients (parabens, polysorbate 80, propylene glycol, benzoates, saccharin sodium, sorbitol and ethanol) [67]. A second strategy should be measuring blood levels and the kinetics of excipients: it has been shown that neonates have a minimal systemic exposure to ethanol following enteral administration of ethanol-containing drugs as part of routine care such as iron and furosemide, although the implication of elevated concentrations in neonates are unclear still now [68].

## 6. TDM and Antibiotics

Antibiotic dosing represents the main strategy to improve clinical outcome in hospitalized neonates [69]. On this regard, 26 different antibiotics have been included in the 100 most used drugs in US NICUs. Furthermore, gentamicin, ampicillin, benzylpenicillin and vancomycin were among the 15 most frequently prescribed drugs in European NICUs, with significant variation in prescription between different gestational age groups of neonates [70,71]. Nevertheless, a lack of neonatal drug usage information on drug labels and the ethical concerns with enrolling children in randomized controlled trials limit the application of proper antibiotic dosing regimen in neonates (Table 1) [72,73].

Both pharmacokinetic and non-pharmacokinetic factors tamper the correlation between dose and drug exposure, limiting to reach the therapeutic target. Application of TDM, along with microbiological results, allows correlating the drug concentration and the bactericidal ability (PK/PD) of antibiotics with the minimal inhibitory concentration (MIC).

Three different PK/PD indices, depending on the antibiotic class, are correlated with the best in vitro antibiotic killing activity and better clinical outcome [74]:

(1) the fraction of time during which the free antibiotic concentration remains above the MIC (%fT > MIC);
(2) the peak concentration of the drug within a well determined range (Cpeak/MIC);
(3) the ratio between the exposure of a given antibiotic (AUC, area under the curve) and the pathogen MIC (AUC/MIC).

Beta-lactams exert their time dependent bactericidal activity through the disruption of pathogens cellular wall. Many of these drugs show limited Phase I hepatic metabolism and are extensively excreted in urine. Dosing schedules of beta-lactams should aim to maintain free plasma concentration over the MIC (fT > MIC) for at least 40–50% of dosing interval. However, severe cases require higher drug exposition (4–5 times above MIC) for the whole interval between doses [73]. Therefore, prolonged or continuous infusions, coupled with a loading dose could ensure a higher level of PK/PD efficacy. Due to the high portion of body water and the reduced renal maturity in neonates, beta-lactams clearance depends primarily on gestational age (GA), postmenstrual age (PMA) and body weight (BW) [75]. These PK features have an impact on dosing guidelines in neonates as stated for penicillin G, cefotaxime and meropenem [76,77,78]. Because of the wide therapeutic range, TDM for beta-lactams is not frequently used in clinical routine and few centers perform beta-lactams monitoring, even in ICU setting [79]. Consensus on toxic effect thresholds has not been defined yet. However, trough concentrations associated with a 50% risk of developing neuro- and nephrotoxicity have been described for piperacillin, meropenem and flucloxacillin in adults [80].

Aminoglycosides (AG) are primarily prescribed to target aerobes Gram-negative pathogens. Due to the huge inter-individual variability in PK and the correlation between dose and side effects, TDM is a standard tool for treatment optimization. AG display concentration-dependent bacterial killing and dose should attain a Cpeak/MIC ratio of 8–10, with a trough concentration (Ctrough) lower than 5 mg/L for amikacin and less than 1 mg/L for gentamicin and tobramycin to avoid oto- or nephro-toxicities [81,82]. Acute kidney injury may occur in one third of children exposed to AG [83,84]. The “extended once daily” dose is more effective than multiple daily dosages and able to reduce side effects and adaptive resistance [27,85,86,87]. Due to the hydrophilic nature, elimination is mainly but not exclusively mediated by glomerular filtration. In addition, as brilliantly defined by Allegaert and van der Anker, these features pose a “catch-22” dosing issue in newborns: due to the higher relative distribution volume in neonates (L/kg, body water content), a higher mg/kg dose is needed, while the reduced glomerular filtration subsequently necessitates a further extended time interval, sometimes beyond 48 h, as validated for an amikacin dosing regimen in neonates [70]. Amikacin is an AG mainly used to treat Gram-negative infections in NICU and appears to be less nephrotoxic than gentamicin and tobramycin [84]. On the other hand, in low birth weight (LBW) infants exposed to amikacin (mean dose of 14 mg/kg), ototoxicity occurred in 4 out of 20 patients. To confirm the TDM significance, the same authors revealed that Ctrough > 10 mg/L was related with hearing and renal dysfunction [83]. Moreover, practicing a clear and updated TDM protocol for gentamycin-exposed newborns improves the attainment of target peak and trough concentrations [88]. NeoFax guidelines suggest different dosing interval based on PMA and subsequently adapted on trough levels at 24 h after the administered dose, with a target less than 1 mcg/mL [82]. In addition, clinicians must remember that AG elimination is affected by nephrotoxic drugs co-administration such as NSAIDs (indomethacin and ibuprofen) and vancomycin [86].

Vancomycin is a glycopeptide antibiotic indicated for methicillin resistant *Staphylococcus aureus* strains (MRSA), vancomycin-intermediate *Staphylococcus aureus* strains (VISA) and *Coagulase Negative Staphylococci* (CoNS) infections in children. Vancomycin works by binding to d-alanyl-d-alanine, a precursor to the cell wall that is crucial for crosslinking peptidoglycan. Its pharmacological action is bactericidal against most Gram-positive species, including streptococci and staphylococci, with the exception of *Enterococcus species*, for which it is bacteriostatic. Based on the recent consensus guideline by IDSA, an AUC guided TDM of vancomycin is recommended for all pediatric groups [72].

Neonate dose recommendations are based on the serum creatinine levels, the postmenstrual age and the bodyweight, ranging from 10 to 20 mg/kg every 8–48 h. A target AUC/MIC ratio between 400 and 600 mcg/mL was extrapolated from in vitro studies or adult data, accounting only for MRSA MIC (<1 mg/L). As a surrogate marker of AUC/MIC, trough concentration is still considered in clinical practice, due to its simpler approach, although it poorly predicts a specific concentration–time profile [89]. Trough levels should be checked just before the fourth dose, when steady-state levels are likely to have been achieved and should be higher than 10 mg/L to prevent the development of resistance. More frequent monitoring may be considered in patients with fluctuating renal function. Nevertheless, the use of a Bayesian dose-optimization software, pairing population PK parameters with the individual observed drug concentration, provides a posteriori value distribution of the patient PK profile. Therefore, blood samples collected within the first 24–48 h can be used to choose the optimal dosing regimen for each patient [90].

CoNS appear to exhibit higher MIC breakpoint than *S. aureus*. Despite this, a study on 152 preterm and term neonates reported a low rate of treatment failure in CoNS group, suggesting that lower AUC/MIC ratio may be appropriate for CoNS mediated late onset infection [91].

## 7. TDM and Antifungals

Antifungal drugs are classified according to their mechanism of action. Polyenes, azoles and allylamines act on the cell membrane of fungi and have ergosterol as a target of pharmacological action; echinocandins (micafungin, caspofungin and anidulafungin) block the fungal cell wall synthesis by inhibiting the enzyme 1,3-beta glucan synthase; and antimetabolites (5-flucytosine and flucytosine) have fungistatic activity by inhibiting the DNA/RNA synthesis.

Some antifungal drugs represent ideal candidates for TDM [92]: itraconazole, voriconazole and posaconazole among azoles and 5-flucytosine among antimetabolites. They are characterized by high inter- and intra-patient variability in blood concentration, absorption, distribution, metabolism, elimination, genetic polymorphism, drug–drug interaction and narrow therapeutic index [93]. PK data for voriconazole and posaconazole already exist for adult patients, but they are not available for pediatric and neonatal patients [94], given the wide differences in volume distribution, clearance and other PK parameters, especially in neonatal age [92]. Small changes in dose could give wide changes in plasma concentration. This variability depends mainly on genetic polymorphism of CYP2 C19 [95]. In relation to their genetic profile and the ability to metabolize the drug, individuals can be classified into three group: poor metabolizers, extensive metabolizers and ultra-rapid metabolizers [26]. However, target voriconazole concentration attainment could be facilitated by dedicated software for dosage individualization. Specifically, through the integration of a population model into a multiple model Bayesian adaptive dosing controller, this tool provides a solid TDM management and dosage individualization in children, using a unique sample, obtained anytime and not necessarily at steady state [96,97]. Lempers et al. in a retrospective analysis of oncology patients focused on the importance of TDM of voriconazole and underlined age as one of the most important factors that influence voriconazole plasma exposure. The authors recruited 21 patients but only three under two years: the drug clearance was much higher in children than adults, and, in the absence of therapeutic monitoring, it was very difficult to ascertain the real exposure of patients to therapeutically effective plasma drug levels [98]. Luong et al., by a meta-analysis, displayed an exposure–response relationship between voriconazole serum concentration and successful outcomes. The authors correlated subtherapeutic voriconazole serum concentrations and development of toxicity: 24 studies were included in the meta-analysis but only three performed only on pediatric patients [99]. Although voriconazole is not recommended in children less than two years and infants, several case reports with an off-label use for *Candida* spp. treatment-resistant or aspergillosis in neonates and infants have been described [100,101,102].

Table 2 summarizes antifungals currently used in neonatal age that are titrated with TDM [103,104].

Fluconazole has a direct correlation between the administered dose and the plasma concentration, so TDM is not routinely required in the clinical practice [26,105]. Nevertheless, fluconazole concentration monitoring may be indicated in neonatal population due to developmental change of drug clearance. Indeed, in neonates, fluconazole half-life changes over the weeks ranging from 73 h for premature infants to 46 h at 12 days of age. Moreover, fluconazole Cl normalized to body surface area in newborns was strongly reduced if compared to adult values [106]. Thus, due to the specific PK features in neonatal population, less frequent dosing should be considered, especially in the first 15 days of life [107]. Several studies have been performed on fluconazole prophylaxis in *Candida* infections in preterm and term neonates [106,108,109,110,111], investigating safety, efficacy, duration and optimal dosing of fluconazole prophylaxis. The shared conclusion is that fluconazole as prophylaxis in neonates is safe and there is no evidence of drug resistance development among fungal species. Nonetheless we need larger studies to expand PK and drug resistance data in this particular population.

The efficacy of echinocandins correlates with the dose and with the dose in 24 h. They require dose adjustment, mainly in pediatric patients. There are no shared indications for TDM in adults, because the predictable response and the low interpatient variability [92]. Micafungin is the only echinocandin approved for small children and neonates. The recommended dose varies from 2 to 10 mg/kg/day. In neonates and small infants, the clearance of micafungin is higher than in adults and children. Liver function and the level of circulating plasma proteins can also influence the free portion of the drug and affect its elimination. Therefore, in the case of administration of micafungin, especially in premature infants, TDM should be regularly performed.

Polyenes, e.g. amphotericin B deoxycholate, bind ergosterol in the fungal cell membrane, depolarize the membrane and cause the formation of pores that increase permeability to proteins and electrolytes, leading to the death of fungal cells. At high concentrations, polyenes bind to cholesterol in mammalian cell membranes, leading to various organ toxicities. Liposomal Amphotericin B is a formulation of amphotericin B that has been linked with liposomes, to reduce side effects of the drug, mainly the nephrotoxicity. Liposomes preferentially attach to the fungal cell wall. The active amphotericin B molecule is released and transfers to the cell membrane, where it can exert its activity, forming pores and leading to ion leakage and cell death. This drug differs in PK parameters from amphotericin B deoxycholate. Despite the extensive use in neonates and premature infants with suspected or proven invasive fungal infection, there are no robust PK data on liposomal amphotericin B in this population and no good information on optimal dosing regimens. Efficacy and safety comparison studies between echinocandins and amphotericin B formulation to treat *Candida* infections in neonates and infants conclude that there are no differences in efficacy and echinocandins give fewer adverse events than amphotericin B formulation [112,113,114]. All these studies explain the importance to further implement PK data availability of these drugs on neonates.

## 8. TDM and Antiepileptic Drugs

Scientific interest in targeted treatment or precision medicine in neonatal seizures is growing [115]. Phenobarbital (PB) and phenytoin (PHT) are confirmed to still be the most commonly used antiepileptic drugs (AEDs) in NICUs [115], although there are concerns about their potential neurotoxicity on the developing brain [116]. In contrast, levetiracetam (LEV) is increasingly prescribed [117], and there have been no reports on LEV neurotoxicity [118]. Table 3 summarizes the pharmacokinetic features of the most used AEDs in newborns, which are mostly administered starting with a loaded dose, followed by maintenance doses [119,120]. Moreover, a combination of two or more AEDs is often required to control neonatal seizures and PK interactions play a key role in determining the clinical effects.

TDM has extensively been used for older drugs such as PB or PHT. Phenobarbital efficacy has been linked to concentrations of 10–40 mg/L; due to the prolonged drug half-life, a routine TDM after few days of treatment with PB is recommended [27]. Phenytoin is used as second-line treatment, with a target PHT level of 10–20 mg/L. This drug is not easy to handle because of risks for cardiac arrhythmias and hypotension, high potential for interactions with other drugs (e.g., PB) and need for frequent blood samples to monitor its levels [121].

Target concentrations of new antiepileptic drugs are still unknown for adults as well as newborns [2]. Levetiracetam has a favorable linear PK, is mainly excreted unchanged by the kidneys and is metabolized via enzymatic hydrolysis by a plasma esterase. The plasma therapeutic range for LEV may vary from 5 to 65 mg/L [118]: in neonates, further studies are needed to establish the target plasma concentration of LEV. Lower clearance of LEV in neonates is caused by a lower GFR and possibly by lower plasma esterase activity [122]. LEV is secreted into saliva and concentrations are similar to plasma unbound levels; therefore, saliva samples could be used in centers where this test is available, although considering eventual contamination of saliva samples when patients are receiving oral doses [118]. LEV undergoes minimal hepatic metabolism and has lower protein binding (<10%) than other AEDs such as phenytoin (<90%), resulting in fewer interactions with other drugs [123].

Intravenous continuous infusions may be indicated when seizures persist under a maximal treatment: therapeutic options include midazolam or lidocaine [124]. Despite pediatricians’ concern on midazolam (MID) use in spontaneously breathing infants, due to the risk of respiratory failure, midazolam may be started with a loading dose and then clinically titrated to effect: its advantages are the rapid metabolic inactivation, clearance and comparatively short elimination half-time [125]. A therapeutic range is not used in the clinical practice. Lidocaine (LID) is not widely used in NICUs, due to the risk of side effects on the heart: a concentration/effect relation seems likely with a plasma concentration of 9 mg/L as a threshold for increased risk of cardiotoxic effects, but this has not been confirmed sufficiently by clinical studies on infants [126]. Recent advances in personalized antiepileptic therapy include the use of carbamazepine (CBZ) in genetic conditions such as benign familial neonatal epilepsy (BFNE): seizures promptly respond to low-dose oral CBZ, even in the case of status epilepticus [127]. TDM could be used while prescribing CBZ: optimum seizure control in patients on monotherapy is most likely to occur at CBZ plasma concentrations of 4–12 mg/L.

Little is still known about the safety of topiramate and bumetanide in neonates [119]: further investigations are needed.

Finally, we should not forget that some rare inborn errors of metabolisms could clinically present with severe seizures that can respond to pharmacological doses of vitamins. Mathew et al. showed how a LC-MS method could simultaneously quantify different biomarkers from a single dried blood spot sample [128] to promptly identify selected vitamin-responsive seizures such as biotinidase deficiency, maple syrup urine disease, methylenetetrahydrofolate reductase deficiency and methyl cobalamin deficiency.

## 9. TDM in Specific Populations

### 9.1. Neonates under Therapeutic Hypothermia

Therapeutic Hypothermia (TH) is now a standard-of-care in term neonates following mild-severe perinatal asphyxia, because of its effects on cerebral metabolism. However, TH also affects cardiac output, renal blood flow and whole metabolic activity: PK and PD changes have been described, with a decreased drug clearance by glomerular filtration [129] and a decreased activity of hepatic cytochrome enzymes [130]. More than half of infants with hypoxic-ischemic encephalopathy (HIE) have neonatal seizures: therefore, the influence of TH on the metabolism of antiepileptic drugs should be carefully considered. Phenobarbital administered to neonates under whole-body TH resulted in higher plasmatic concentrations and longer half-lives than expected in their normothermic peers [131], although no clinically relevant effect of TH on phenobarbital were identified with an observed responsiveness of 66% in a Dutch study [132]. According to the results of the Pharma-Cool study, TH did not influence clearance of PB or MID in (near-)term neonates with HIE but a PB dose of 30 mg/kg (instead of 20 mg/kg) was advised to reach therapeutic concentrations [46]. Furthermore, PB co-administration significantly increased MID clearance and a 50% lower MID maintenance dose might be more appropriate during the first days after birth. Clinicians should also pay attention during rewarming, where on the contrary metabolism is increased and drug elimination could lead to significative reduction in drugs concentrations [130]. Similar concerns include antibiotic administration, frequently administered during TH. Amikacin clearance was decreased to 40.6% in these neonates and a 12-h increase in the amikacin dosing interval was proposed to correct for the reduced clearance [133]. A decreased clearance of gentamycin during TH was confirmed by a meta-analysis of eight studies: modified gentamicin dosing regimens with 36-h intervals are required to yield safe and effective exposures [134]. Considering reduced drug clearance, Cies et al. suggested that dosing regimens of ampicillins 25–50 mg/kg/day were sufficient to protect against susceptible bacteria in neonates with HIE [135].

### 9.2. Preterm Infants

Preterm neonates need pharmacovigilance strategies that should be tailored to different stages of maturation and further studies on differences in the PK of drugs in this population compared to term infants and older children are needed. They have relatively reduced body fat stores and a TBW that is even higher compared with that of their full-term peers [11]. They have a reduced number of nephrons at birth and are often exposed to nephrotoxic drugs (i.e., aminoglycosides), while nephrogenesis is still ongoing [136]. TDM is advised for treatment beyond three days [137]. Preterm infants are at higher risk of overdosing due to the immature renal function [90]: acute kidney injury is considered an independent predictor of mortality, especially when the birthweight is less than 1500 g, and different biomarkers and definitions have been identified for the early detection of renal damage in these neonates, however they need validation [138]. This is important especially for drugs exclusively eliminated by renal excretion such as glycopeptides and aminoglycosides. Small for gestational age (SGA) infants show a reduction in the clearance of vancomycin and amikacin up to 16% when compared to infants with adequate weight at birth [139].

Furthermore, some drugs such as betamethasone and indomethacin, when administered prenatally to preterm infants, alter their post-natal renal maturation [140].

Immaturity of hepatic metabolism also plays a key role in PK of preterm infants, as in the case of phenobarbital: the half-life is longer in preterm than in term infants due to immaturity of PB metabolism. Furthermore, the Vd is about double in infants than in adults, likely because the unbound fraction of PB is greater in infants [141].

Another drug often used in preterm infants is acetaminophen, which facilitates closure of significant ductus arteriosus via an alternate pathway of prostaglandin inhibition: neonates have an acetaminophen clearance/kg similar to that of adults. However, a relatively lower glucuronidation (that increases with GA) could be observed, with a consequent oxidation of a minor fraction (5–10%) to a highly reactive hepatotoxic metabolite (*N*-acetyl-p-benzoquinone imine). Therefore, preterm infants (GA 24–32 weeks) should be carefully monitored for toxicity even after a single high dose of acetaminophen (10–20 mg/kg) [142].

Extremely LBW infants also have an increased risk of developing ototoxicity, due to a delayed amikacin excretion: administered doses should be carefully titrated according GA, birth weight and creatinine values [83].

Few data are available for use of antifungal agents in premature and LBW infants: micafungin is the most frequently used in newborns suffering from invasive candidiasis. It has been proposed that neonates and very low birth weight neonates may require higher weight-based doses (up to 15 mg/kg/day) of micafungin, in line with allometric scaling of plasma clearance to achieve exposures bioequivalent to those in adults [143]. Our group demonstrated that higher weight-based doses of micafungin were generally well tolerated in preterm neonates and infants with systemic candidiasis (three of whom had meningitis) and achieved pharmacokinetic profiles predictive of an effect: the safety of micafungin was assessed by measurement of the levels of liver and kidney function biomarkers [38].

Use of mother’s milk or formula is also one of the covariates that affect drug metabolism: formula feeding accelerates maturation of CYP3A4 or CYP1A2 [144]. Furthermore, in preterm infants, it may be difficult to distinguish patients with the poor metabolizer genotype from those with underdeveloped CYP2C19 enzymes, as confirmed in a study on metabolism of proton pump inhibitor pantoprazole [145].

As the need of frequent sampling limits the routine use of TDM in preterm newborns, the availability of microsampling techniques (e.g., dried blood spot, DBS) is crucial to study the relationship between administered dose and plasmatic levels, avoiding anemia related to repeated blood pick up [31].

Furthermore, some drugs have a wide therapeutic range, such as caffeine: a therapeutic level of caffeine is considered between 5 and 25 mg/L, while toxic levels are reached with >40–50 mg/L [146]. A retrospective study on infants born less than 29 weeks GA demonstrated that those with an average caffeine concentration higher than 14.5 mg/L had significantly lower incidence of chronic lung disease and patent ductus arteriosus, fewer days on mechanical ventilation and oxygen, less need for diuretics and lower length of stay [147]. If these findings were confirmed prospectively, it could become useful to introduce TDM of caffeine in routine practice, avoiding reported neurologic side effects of high doses [148].

### 9.3. Anticancer Drugs

Anticancer drugs are difficult to administer in neonates because doses are usually tailored on birth weight or body surface area (BSA). A first limitation comes from their growth velocity: weight increases more than 10-fold between birth and 12 years. However, standard BSA-based chemotherapy doses that are tolerable in children and adolescents are proved to be too toxic for infants (≤12 months of age) and doses are thus more based on toxicity rather than on a therapeutic effect [149].

Therefore, TDM could be considered a useful method to guide treatment but few PK data are still available for these infants: to date, few case studies on treatment with methotrexate, carboplatin, cisplatin, vincristine and etoposide are available [150,151,152].

While it may be unrealistic to design large prospective clinical trials to generate data in large numbers of infants with different types of tumors, an empirical temporary solution has been provided by Children’s Oncology Group Task Force of Philadelphia, which suggested a BSA-based dose banding incorporated into dosing tables for conventional anticancer drugs for infants and children with a BSA < 0.6 m^2^, which is reached at about 36 months [149].

### 9.4. Extracorporeal Life Support

Extracorporeal membrane oxygenation (ECMO) is a life-saving therapy even in neonates with potentially reversible neonatal cardiac and/or respiratory failure, whose prognosis would otherwise be dismal. Continuous renal replacement therapy (CRRT) remains the most common dialysis modality for acutely ill children. The combination of ECMO and CRRT seems to be a good method for treating ECMO patients who have developed acute kidney injury [153]. These patients are daily treated with many different drugs: these patients receive heparin to avoid clotting, sedatives and analgesic drugs for sedation and antibiotics, antivirals or antifungals to treat eventual infections.

Pharmacokinetic changes in critically ill ECMO patients are more reflective of critical illness in itself rather than the device. However, ECMO can alter the drugs PK within two main mechanisms: (1) sequestration of the drug to the circuit; and (2) rapid increase in Vd with or without changes in the drug Cl at start of ECMO as a result of hemodilution [154]. 

Routine therapeutic drug monitoring (TDM) in this critical population would be advantageous to prevent side effects or sub-optimal dosing during ECMO but little is still known [155]. Multicentric prospective PK studies in pediatric ECMO patients will let us acquire more data in so critically ill patients. Meanwhile, physiologically-based pharmacokinetic modeling (PBPK) is emerging as an alternative approach to support clinicians in every-day ICU setting during extracorporeal life support [156].

## 10. Potential Future Indications

TDM could be used in the neonates as a diagnostic tool for clinical syndromes after fetal and neonatal exposure through breastfeeding to drugs following maternal use (e.g., lithium, antidepressants, opioid analgesics and anti-epileptics such as lamotrigine).

Artificial neural network (ANN) modeling for neonates could become a promising approach to predicting drug concentrations whenever drawing blood samples from critically ill neonates and infants for TDM is particularly challenging [157].

## Figures and Tables

**Table 1:** Pharmacokinetics of most common antibiotics titrated with TDM in newborns (modified from Rybak et al. [72] and Huttner et al. [73]). iv, intravenous.

Antibiotics	Mechanism of Action	Dose	Metabolism	Excretion	Fraction Unbound (%)	Half-Life (h)
**Vancomycin**	Glycopeptide: inhibits proper cell wall synthesis in Gram-positive bacteria	10-20 mg/kg/dose intermittent iv infusion with a different 8–48 h interval according PMA or 50 mg/kg/day continuous iv infusion according to serum creatinine and corrected GA	Excreted unchanged	Renal (>90%)	70–90	2–10
**Gentamicin**	Aminoglycoside: binds 30S subunit of the bacterial ribosome	4–5 mg/kg/dose with a different 24–48 h interval according GA/PMA	No metabolism	Renal (90%)	90	7–14 (if GA ≤ 30 w); 4–7 (if term)
**Amikacin**	Aminoglycoside: binds 30S subunit of the bacterial ribosome	15–18 mg/kg/dose with a different 24–48 h interval according PMA	No metabolism	Renal (90%)	90	7–14 (if GA ≤ 30 w); 4–7 (if term)

**Table 2:** Pharmacokinetics of most common antifungals titrated with TDM in newborns (modified from Roberts et al. [103] and Bersani et al. [104]). iv, intravenous.

Antifungals	Mechanism of Action	Dose	Metabolism	Excretion	Fraction Unbound (%)	Half-Life (h)
Micafungin	Echinocandin: inhibition of beta-1,3-glucan	4–15 mg/kg/day iv (in a hour)	Hepatic	Hepatic (bile) (77%), renal (12%)	1	8
Fluconazole	Triazole: inhibition of lanosterol 14α-demethylase	12 mg/kg/day (for therapy); 3–6 mg/kg/day 2 or 3 times in a week (for prophylaxis)	Hepatic (minimal)	Renal (80%)	89	15–25 (in preterms < 29 weeks: 73.6 h after 24 h and 46.6 h at 12 days)
Liposomal Amphotericin B	Polyene: binding with sterols in the fungal cell membrane	3–7 mg/kg/day	Unknown	Unknown	5	7

**Table 3:** Pharmacokinetics of most common antifungals titrated with TDM in newborns (modified from Donovan et al. [119] and Patsalos [120]). iv, intravenous.

Antiepileptic Drug	Mechanism of Action	Loading Dose	Maintenance Dose	Metabolism	Excretion	Fraction Unbound (%)	Half-Life (h)
Phenobarbital	GABA-A agonist	20 mg/kg in 20 min iv	5 mg/kg/day	Hepatic	Renal (of whom 25–50% unchanged)	57–64	73.9–154.5
Phenytoin	Blockade of voltage-gated sodium channels	20 mg/kg in 30 min iv	5 mg/kg/day	Hepatic	Renal (of whom up to 5% unchanged)	17.2–22.4	Week 1: 9.1–32.3 Week 2–4: 4.1–11.1
Levetiracetam	Binding to neuronal SV2a receptor	10–50 mg/kg iv	10–80 mg/kg/day	Minimal hepatic	Renal (of whom up to 66% unchanged)	~100	Day 1: 11.4–25.6 Day 7: 7–11
Midazolam	GABA-A agonist	0.05 mg/kg in 10 min iv	0.15 mg/kg/h	Hepatic	Renal (~70%), Feces (~30%)	3.1	6.5–12
Lidocaine	Blockade of fast voltage-gated sodium channels	2 mg/kg iv	5–7 mg/kg/h for 4 h; 2.5–3.5 mg/kg/h for 6–12 h;1.25–1.75 g/kg/h for 12 h (to reduce during hypothermia)	Hepatic	Renal	20–40	5.2–5.4
Carbamazepine	Blockade of voltage-gated sodium channels	Available only enteral form (oral bioavailability 75–85%)	10 mg/kg/day	Hepatic	Renal (~70%), Feces (~30%)	25	10–13

## Abbreviations

%fT > MIC	Fraction of time above the MIC
ADIs	Accepted daily intakes
AEDs	Antiepileptic drugs
AG	Aminoglycosides
AUC	Area under the curve
BSA	Body surface area
CATCH-22	Paradoxical situation (the term was coined by Joseph Heller for his novel Catch-22)
CBZ	Carbamazepine
Cl	Clearance
CMS	Capillary microsampling
CONS	Coagulase negative staphylococci
Cpeak	Drug peak concentration
CrCl	Creatinine clearance
Ctrough	Trough concentration
CYP	Cytochrome
DBS	Dried blood spot
ELBW	Extremely Low Birth Weight
ESNEE	European Study of Neonatal Exposure to Excipients
F	Bioavailability
GA	Gestational Age
GFR	Glomerular filtration rate
GC	Gas chromatography
HIE	Hypoxic-ischemic encephalopathy
LBW	Low Birth Weight
LC	Liquid chromatography
LC-MS/MS	Mass spectrometry coupled with high performance liquid chromatography
LEV	Levetiracetam
LID	Lidocaine
MIC	Minimal inhibitory concentration
MID	Midazolam
MRSA	*Methicillin Resistant Staphylococcus aureus*
MS	Mass spectrometry
NICUs	Neonatal intensive care units
PB	Phenobarbital
PD	Pharmacodynamics
PHT	Phenytoin
PK	Pharmacokinetics
PMA	Postmenstrual age
STEP	Safety and toxicity of excipients for paediatrics
TDM	Therapeutic drug monitoring
TH	Therapeutic hypothermia
VAMS	Volumetric absorptive microsampling
Vd	Volume of distribution
VISA	*Vancomycin-Intermediate Staphylococcus aureus*
